# An alternative method to characterize the surface urban heat island

**DOI:** 10.1007/s00484-014-0902-9

**Published:** 2014-09-19

**Authors:** Philippe Martin, Yves Baudouin, Philippe Gachon

**Affiliations:** 1 Meteorological Service of Canada, Environment Canada, Montréal, Québec Canada; 2 Department of Geography, University of Québec at Montreal (UQAM), Montréal, Canada; 3 Canadian Centre for Climate Modelling and Analysis (CCCMA), Climate Research Division, Environment Canada, Montréal, Canada; 4 ESCER (Étude et Simulation du Climat à l’Échelle Régionale) Centre, University of Québec at Montréal, Montréal, Canada

**Keywords:** Urban, Rural, Surface urban heat island, Intra-urban, Landsat imagery

## Abstract

An urban heat island (UHI) is a relative measure defined as a metropolitan area that is warmer than the surrounding suburban or rural areas. The UHI nomenclature includes a surface urban heat island (SUHI) definition that describes the land surface temperature (LST) differences between urban and suburban areas. The complexity involved in selecting an urban core and external thermal reference for estimating the magnitude of a UHI led us to develop a new definition of SUHIs that excludes any rural comparison. The thermal reference of these newly defined surface intra-urban heat islands (SIUHIs) is based on various temperature thresholds above the spatial average of LSTs within the city’s administrative limits. A time series of images from Landsat Thematic Mapper (TM) and Enhanced Thematic Mapper Plus (ETM+) from 1984 to 2011 was used to estimate the LST over the warm season in Montreal, Québec, Canada. Different SIUHI categories were analyzed in consideration of the global solar radiation (GSR) conditions that prevailed before each acquisition date of the Landsat images. The results show that the cumulative GSR observed 24 to 48 h prior to the satellite overpass is significantly linked with the occurrence of the highest SIUHI categories (thresholds of +3 to +7 °C above the mean spatial LST within Montreal city). The highest correlation (≈0.8) is obtained between a pixel-based temperature that is 6 °C hotter than the city’s mean LST (SIUHI + 6) after only 24 h of cumulative GSR. SIUHI + 6 can then be used as a thermal threshold that characterizes hotspots within the city. This identification approach can be viewed as a useful criterion or as an initial step toward the development of heat health watch and warning system (HHWWS), especially during the occurrence of severe heat spells across urban areas.

## Introduction

Urban city centers tend to have higher solar radiation absorption and greater thermal capacity and conductivity than the surrounding area (Weng 2001). These modified thermal conditions can cause the local air and surface temperatures to increase by several degrees Celsius over the simultaneous temperatures of the surrounding rural areas (Oke 1982). These urban heat islands (UHIs) result partly from the physical properties of the urban landscape and partly from the release of heat into the environment by the use of energy for human activities. With an increasing area of impervious and man-made non-vegetated surfaces within major metropolitan cities (i.e., through the use of paving or building materials), more of the incoming energy is converted to surface-sensible heat fluxes, thus reducing a given city’s ability to shed its excessive heat by latent heat exchange (Oke 1987). The occurrence and intensity or severity of heat spells has been linked to the UHI effect (Clarke 1972; Rooney et al. 1998). During hot-weather events, UHIs exacerbate thermal stress on the most vulnerable and at-risk people, particularly those with social or physical vulnerability (Kovats and Hajat 2008; Smargiassi et al. 2009).

**Fig. 1:**
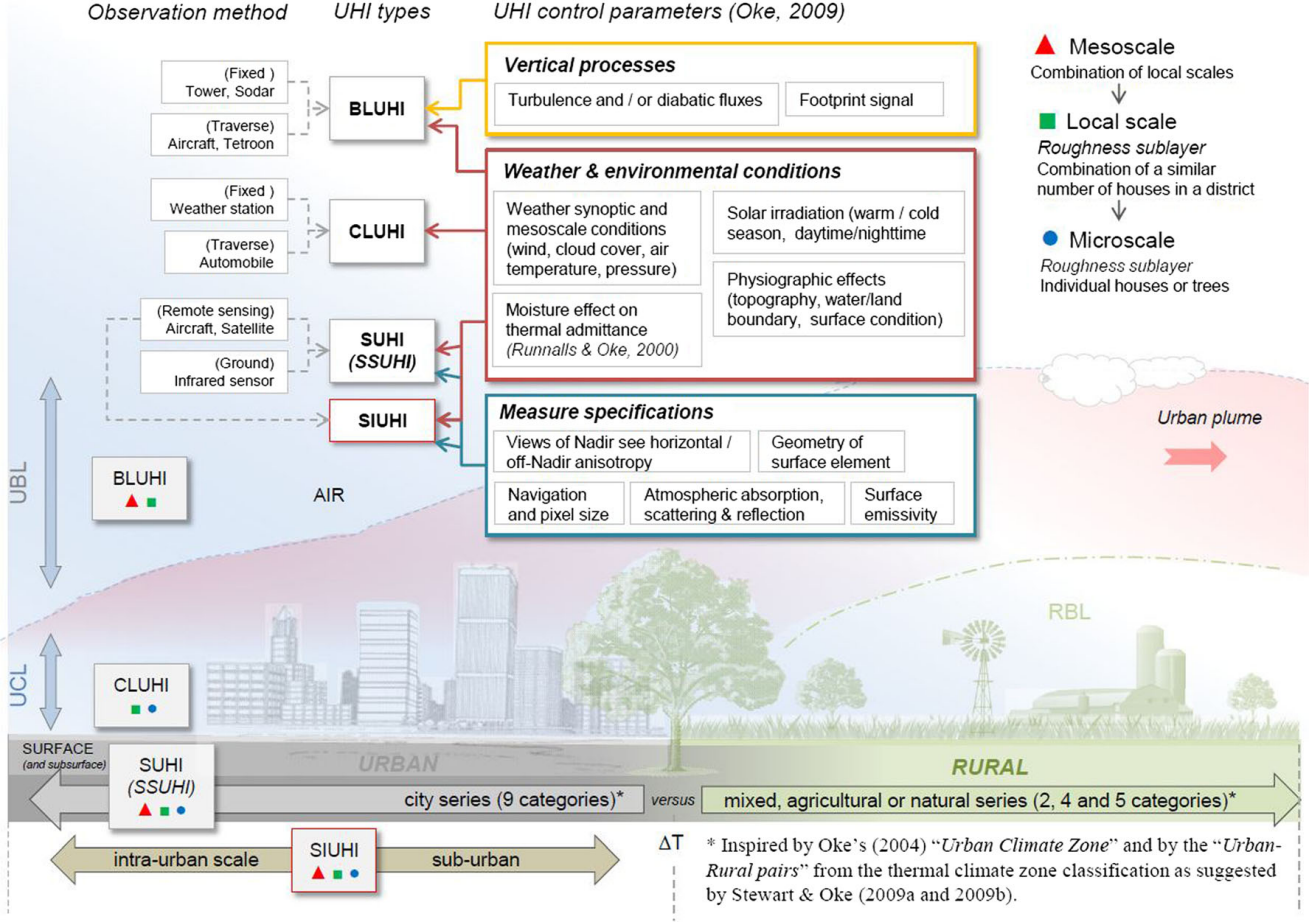
Conceptual diagram of the UHI phenomenon: description of different scales, observation methods, and control parameters that will influence the estimated UHI magnitude (mainly inspired by Oke 1997, 2009; Voogt and Oke 2003; Stewart and Oke 2006, 2009a, b ). All acronyms are defined in the glossary

The original distinction between atmospheric heat islands (cf. Fig. 1) of Oke (1976) may be defined for the urban canopy layer (UCL) or the canopy-layer urban heat island (CLUHI is defined as the area from the surface to approximately the mean building height) and the urban boundary layer (UBL), the layer above the UCL that is influenced by the underlying urban surface (e.g., Voogt and Oke 2003). Both have found expression in an air temperature excess at low levels, while surface or skin (including grass, roofs, trees, and roads) temperature UHIs are detected through the use of airborne or satellite remote sensing. Surface urban heat islands (SUHIs) are observed through the spatial patterns of upwelling thermal radiance received by a remote sensor (Voogt and Oke 2003).

Over the last three decades, medium-resolution thermal infrared imagery and data, such as those available from Landsat Thematic Mapper and Enhanced Thematic Mapper Plus (TM/ETM+), have been extensively employed (120 and 60 m, respectively, for the thermal band—however, all bands are resampled with 30-m resolution before being provided as a final product by the US Geological Survey (USGS)). These satellite data are appropriate for studying intra-urban temperature variations and relating them to surface cover characteristics (Hafner and Kidder 1999; Kim 1992; Parlow 1999; Rigo et al. 2003; Streutker 2002). Carnahan and Larson (1990) used Landsat TM data to analyze thermal conditions in the Indianapolis metropolitan area (Indiana) and found an urban–rural radiant temperature difference of 6.4 °C in the daytime (heat sink). Using Landsat’s two sensors, Xian and Crane (2006) evaluated urban thermal characteristics in the Tampa Bay watershed (Florida, USA) and Las Vegas Valley (Nevada, USA) and found that Tampa Bay has a daytime heating effect (heat source), whereas Las Vegas exhibits a daytime cooling effect (heat sink). This is due to the expected moisture status of rural areas; where rural areas (Las Vegas) are very dry, more rapid morning heating could lead to the apparent presence of a surface cool island, while a wet rural area (Tampa Bay) would be slower to warm than urban areas. Hence, the surroundings of a city, which are used as thermal references for evaluating the UHI magnitude, require as much consideration as urban city centers. Other research across the globe (Jiang et al. 2006; Faris and Sudhakar Reddy 2010; Lee et al. 2011) also confirms the potential of Landsat-derived land surface temperatures (LSTs) for representing the complexity of urban thermal properties.

Despite the fact that numerous studies have attempted to systematize the definition of a UHI, it constitutes a relative measure that varies locally according to built-up density, the area’s physiographic features, and meteorological conditions that occur both before and during the formation of the UHI. There is no specific thermal threshold to define the term heat islands, as the temperature difference within the islands varies as well (Imhoff et al. 2010; Shepherd 2006). In a survey of 189 heat island studies conducted between 1950 and 2007, Stewart (2011) found that 45 % of the reported UHI magnitudes show a poor understanding of experimental methods due to lack of control over the confounding effects of weather, relief, and time or because measurement sites do not properly represent their local surroundings. Lowry (1977) noted that, ideally, the effects of urban influences should only be assessed using measurements if pre-urban observations are available, i.e., observations made before the human disturbance of the land. He pointed out that some areas outside an urban area are influenced by the city (e.g., by a plume of warmer air advected downwind from the city toward rural areas), whereas other areas outside the city remain unaffected. While both types of rural areas may be in zones with anthropogenic influences (e.g., zones in which agriculture is present), these zones constitute non-stationary conditions in their ability to define appropriate surrounding heat island magnitudes, with strong heterogeneous behaviors over space and time.

Historically, the definition of a UHI was built on a delicate concept that requires this rural or non-urban reference; furthermore, this was before the availability of satellite technology. The current urban–rural contrast is different from the pre-urban versus urban dichotomy noted by Lowry (1977). Rural areas are zones that are not urbanized but in which the land has nevertheless been disturbed by humans. Furthermore, a non-urban reference can be located in a different biome than the one in which the city that it references was built. Moreover, in terms of surface energy exchange, the thermal reference is continuously changing from 1 week to the next during the warm season as the farmers redesign the landscape.

Stewart and Oke (2009a, b) proposed a new global framework for heat island observations inspired by the urban climate zones (UCZs) of Oke (2004). They suggested classifying measurement sites by thermal climate zones (TCZs) and redefining UHI magnitude by inter-zone temperature difference. The classification includes nine TCZs in the city series (from open grounds to modern core), versus four TCZs in the agricultural series, five in the natural series, and two in the mixed series. This worldwide standardized communication tool has been developed to allow for the public comparison of UHI magnitudes. Such public comparison is possible because UHI magnitudes are now more objectively defined, measured, and reported using inter-zone temperature differences instead of arbitrary urban–rural differences.

However, these thermal climate zones are not easy to use in operational modes and are still too complex to be incorporated in risk management or alert system development where parsimonious, reproducible, and practical thermal references are needed (in terms of occurrence and threshold). In the same overpass, a satellite image can include different city series as well as natural, agricultural, or mixed series in which vegetation may be present in various stages of evolution. Thus, urban–rural pairs become complex to define and constitute a relative measure specific to the overpass time. In this context, the main objective of the present study is to better characterize an SUHI in terms of temperature exceedance level or threshold with respect to a spatial reference and ambient (or prevalent) meteorological conditions. This clarification is needed to improve the operational capacity of meteorological services, such as those at Environment Canada, to thereby better define alerts during hot spells in the summer and help to determinate hotspots within the city (or surface intra-UHI, i.e., SIUHI) where most people live, including vulnerable ones. The study region is the Montreal Metropolitan area located in Quebec, the second largest and populous city in Canada following Toronto. The first step consists of identifying the intra-urban temperature difference that defines an SIUHI using a time series of satellite images obtained from Landsat TM and ETM+ over the 1984–2011 period. This is accomplished by considering the solar radiation conditions, and other pertinent meteorological parameters if needed, that prevailed before and during the formation of the SIUHI.

Because it is the people living in urban areas (and not those in rural zones) who are exposed to UHIs, we have excluded rural thermal references and focused our attention on SIUHIs within city boundaries, as presented in Fig. 1. For each satellite image, the mean surface temperature of the urban core then becomes the reference. Figure 1 summarizes the conventional urban–rural comparison according to the classification of Stewart and Oke (2006, 2009a, b), as well as our SIUHI definition in the context of previously defined UHIs. The different vertical and horizontal scales that define a UHI are also represented (mainly inspired by Oke 1997; Voogt and Oke 2003), along with the appropriate observation methods (inspired by Oke 2009) and their control parameters (inspired by Runnalls and Oke 2000; Oke 2009).

The paper is organized as follows: The second section presents the data, study area, and methods; the third section presents the results; the fourth section discusses the main implications of the results and how they contribute to the improvement of Montreal’s heat alert system; and the fifth section includes a summary and concluding remarks.

## Data and methods

### Study area

**Fig. 2:**
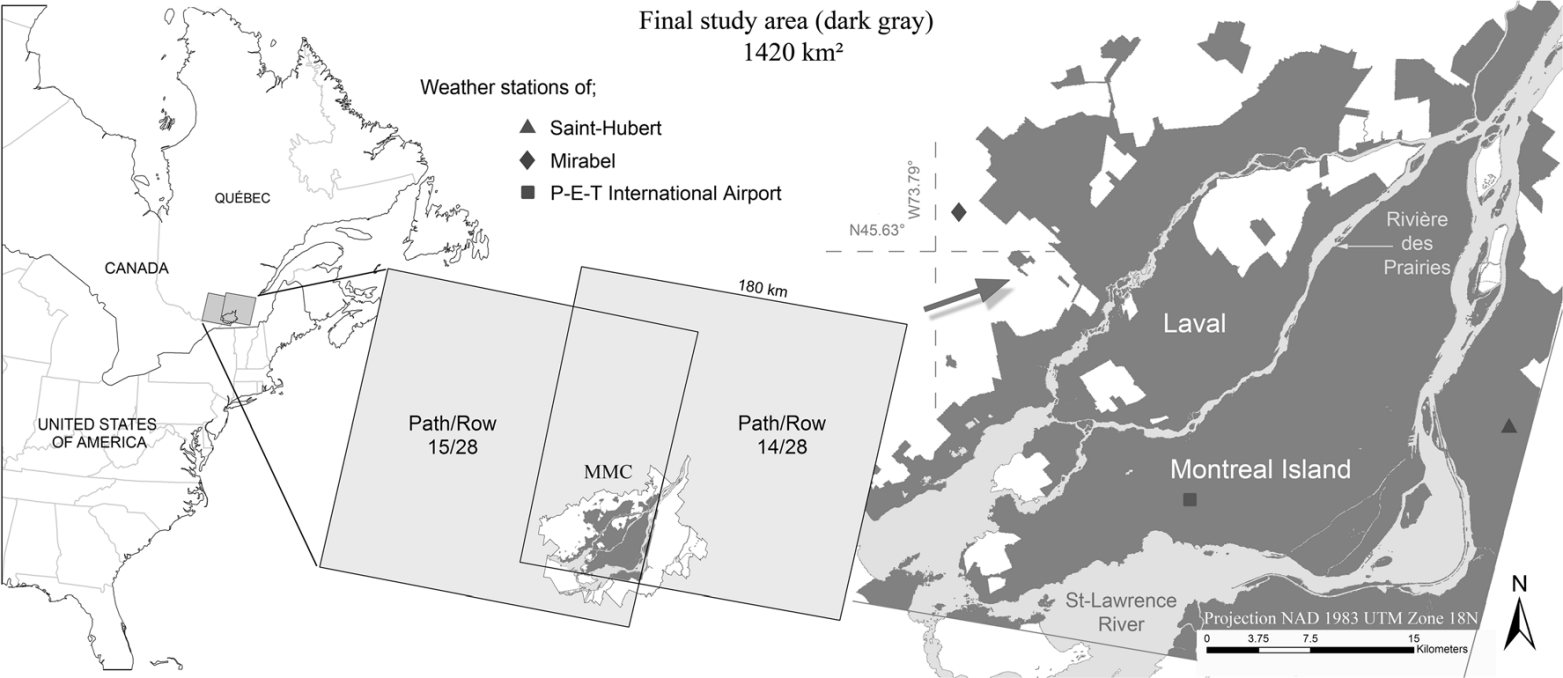
Study area delimited by the extent of the Landsat imageries over the MMC; agricultural and water surfaces are excluded

The study area covers the Montreal Metropolitan Community (MMC) located in the province of Québec, Canada, (at approximately 45° 20′ N/45° 45′ N latitudes and 74° 04′ W/73° 25′ W longitudes). It includes a diversity of land use/land cover classes interspersed with rivers and lakes and with elevations ranging from 6 to 233 m. However, because we used a total of 12 Landsat multispectral images covering different areas with various extents of overlap, it was necessary to create a common spatial frame within the administrative boundary of the city. Therefore, Landsat’s cover in the southern portion of the province (path/row 014/028 and 015/028) excludes either the eastern or southern part of the MMC (Fig. 2, middle). After initially cropping the images, we used a geographical information system (GIS) to remove pixel values over both rivers and over all agriculturally active areas located within the MMC (Fig. 2, right). This procedure was conducted to help or improve the identification and effectiveness of the SIUHI analysis over the MMC urban area. The land mask shown in Fig. 2 defines the study area and was used for each image.

### Image screening

Detailed attention was devoted to the selection of 12 satellite images with particular or consistent meteorological considerations, i.e., images obtained under clear skies and anticyclonic conditions. More precisely, the value of the cloud cover quadrant over the MMC for each image selected was equal to or less than 3 % (lower right quadrant for path/row 015/028 and lower left quadrant for 014/028; see Table 1). To preserve the best consistency among images with respect to the stage of vegetation development and the amount of sunshine, images were selected spanning the months of June and July. Image extents, dates, times, and cloud cover percentage are provided in Table 1. To obtain image-specific measures of ambient temperature, mean sea level pressure, wind speed and direction, and relative humidity for the date and hour of each image, five hourly meteorological parameters and atmospheric variables were averaged (from approximately 10:00 a.m. to 11:00 a.m., which corresponds to the local time of Landsat overpasses).

**Table 1:** Satellite image data summary and meteorological conditions

Sensor (Landsat)	Satellite’s overpass			Cloud cover quadrant lower right/left (%)	Average between 10:00 a.m. and 11:00 a.m.				
	Extent (path/row)	Date	Hour (a.m.)		Mean sea level pressure (kPa)	Wind speed (km/h)	Wind direction	Air temp. (°C)	Relative humidity (%)
Five TM	015/028	July 10, 1984	10:12	0.01	100.81	9	SW	23.5	53.5
Five TM	015/028	June 17, 1987	10:08	0.01	101.52	12	NW	19.1	61.6
Five TM	014/028	July 17, 1989	10:05	0.01	101.15	8.5	SW	24.2	54.5
Five TM	014/028	July 25, 1992	10:00	0.01	101.63	10	SW	23.2	64.5
Five TM	014/028	June 29, 1994	09:56	3.02	100.41	15.5	SE	24.8	67.5
Five TM	015/028	June 28, 1997	10:15	0.01	101.24	9	SW	26.6	60.5
Five TM	014/028	June 11, 1999	10:16	0.08	102.09	11	SW	24.3	59
Seven ETM+	014/028	June 08, 2001	10:27	0.01	100.62	15	SW	20.2	42.5
Five TM	015/028	July 15, 2003	10:20	0.2	101.38	9	SW	25.7	50
Five TM	014/028	June 27, 2005	10:25	0.01	101.79	7	SE	27.2	56
Five TM	014/028	July 05, 2008	10:25	0.01	101.24	13	SW	23.4	50
Five TM	014/028	July 14, 2011	10:27	0.01	101.36	10	SW	22.8	60

Times are given using Eastern Standard Time, i.e., local time, and data are from the Environment Canada weather station at Montreal’s Pierre Elliott Trudeau (P-E-T) International Airport (see its location in Fig. 2); for the 12 dates considered between 1984 and 2011

### The Landsat (TM and ETM+) LST calculation algorithm

**Fig. 3:**
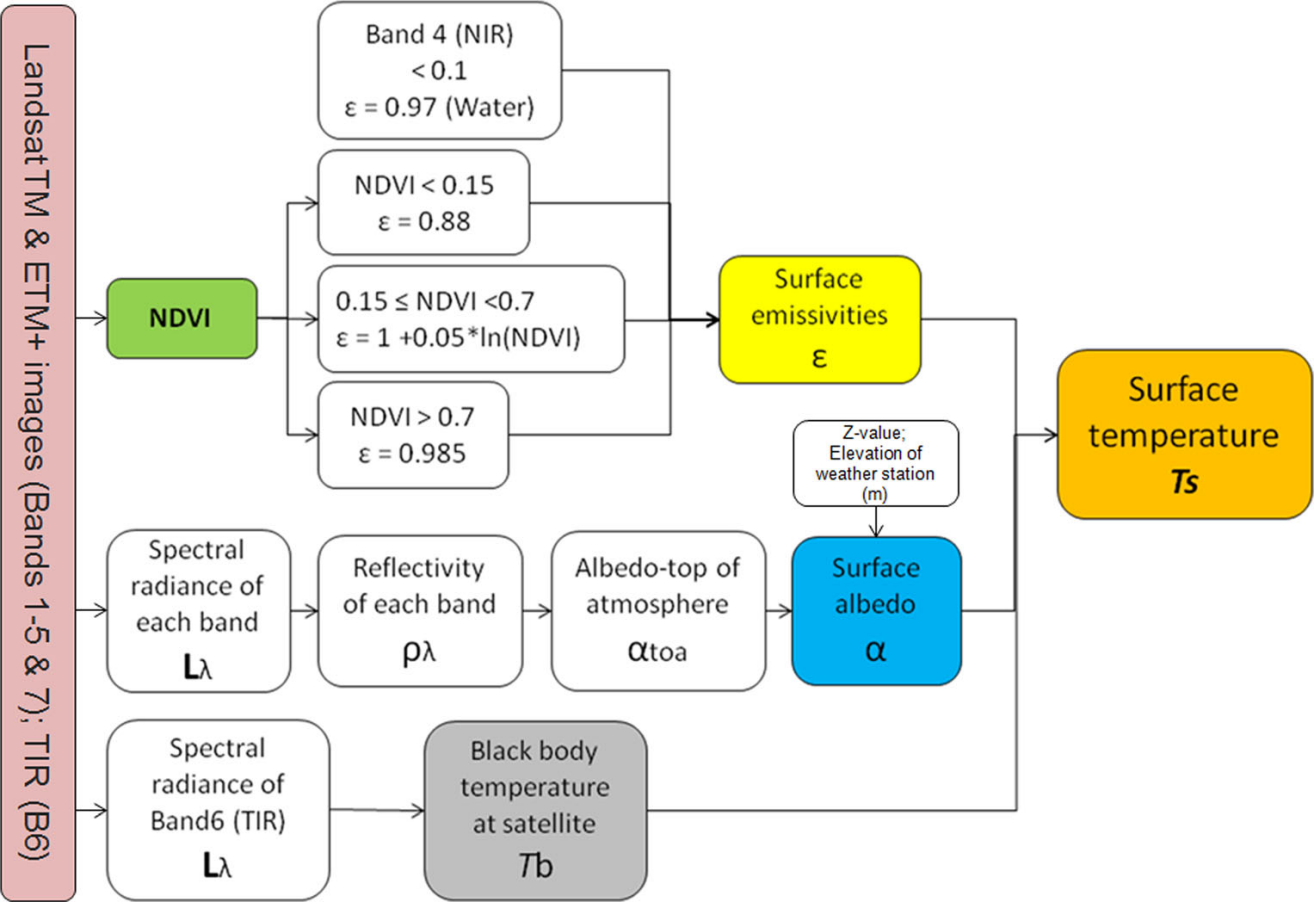
Flowchart for the LST calculation, which was developed into an automated model in a GIS tool

LST is calculated using an equation that includes the blackbody temperature on board the satellite (obtained from band 6 of Landsat), the surface albedo, and the surface emissivity derived from the Normalized Difference Vegetation Index (NDVI), which is calculated using all the other bands of Landsat (see Fig. 3).

The complete procedure is presented in the following five steps (Allen et al. in SEBAL,^1^ ^2002^):

1. Surface albedo (see Fig. 3) is defined as the ratio of the reflected radiation to the incident shortwave radiation. It is computed following the suggested four steps:
  - The spectral radiance for each band (*L*_*λ*_) is computed using the following equation given for Landsat 5 and 7:
    1 $$ {\mathrm{L}}_{\lambda }=\left(\frac{Lmax- Lmin}{QCALmax- QCALmin}\right)\times \left( DN- QCALmin\right)+ Lmin $$
    where *DN* is the digital number of each pixel, *Lmax* and *Lmin* are the calibration constants (see Tables 6.1 and 6.2 in SEBAL (Allen et al. 2002), Appendix 6); *QCALmax* and *QCALmin* are the highest and lowest range of values for rescaled radiance in *DN*. The units for *L*_*λ*_ are W/m^2^/sr/μm.
  - The reflectivity for each band (*ρ*_*λ*_) is then computed using the following equation:
    2 $$ {\rho}_{\lambda }=\frac{\pi \cdot L\lambda\ }{ESUN\uplambda \cdot \kern0.5em  \cos \theta \cdot {d}_r\ } $$
    where *L*_*λ*_ is the spectral radiance for each band computed in (a), ESUN_*λ*_ is the mean solar exo-atmospheric irradiance for each band (W/m^2^/μm), cos *θ* is the cosine of the solar incidence angle (from nadir), and *d*_*r*_ is the inverse squared relative earth–sun distance (values for ESUN_*λ*_ are given in Table 6.3 of SEBAL (Allen et al. 2002), Appendix 6). Cosine *θ* is computed using the header file data on sun elevation angle (*β*) where *θ* = (90° − *β*). The term *d*_*r*_ is defined as 1/*d*_e–s_^2^ where *d*_e–s_ is the relative distance between the earth and the sun in astronomical units. *d*_*r*_ is computed using the following equation by Duffie and Beckman (1980):
    3 $$ {d}_r=1+0.033\  \cos \left( DOY\frac{2\pi }{365}\right) $$
    where DOY is the sequential day of the year (or Julian day), and the angle (DOY × 2*π*/365) is in radians.
  - The albedo at the top of the atmosphere (*α*_toa_) is then computed as follows:
    4 $$ {\alpha}_{\mathrm{toa}}={\displaystyle \sum}\left(\omega \lambda \times \rho \lambda \right) $$
    where *ρ*_*λ*_ is the reflectivity computed in (b), and *ω*_*λ*_ is a weighting coefficient for each band (see Table 2; obtained by SEBAL (Allen et al. 2002), Table 6.4, Appendix 6).

**Table 2:** Bands weighting coefficients, *ω*_*λ*_

	Band 1	Band 2	Band 3	Band 4	Band 5	Band 6	Band 7
Landsat 5	0.293	0.274	0.233	0.157	0.033		0.011
Landsat 7	0.293	0.274	0.231	0.156	0.034		0.012

See Table 6.4 of SEBAL (Allen et al. 2002), Appendix 6
    The final step is to compute the surface albedo (*α*) by correcting the *α*_toa_ for atmospheric transmissivity:
    5 $$ \alpha =\frac{\alpha \mathrm{toa}-\alpha \mathrm{path}\ \mathrm{radiance}}{\tau \mathrm{sw}\kern.1em 2\kern1.5em } $$
    where *α*_path radiance_ is the average portion of the incoming solar radiation (range between 0.025 and 0.04) across all bands that is back-scattered to the satellite before it reaches the Earth’s surface (we chose the value of 0.03 based on SEBAL (Allen et al. 2002)), and *τ*_sw_ is the atmospheric transmissivity which is defined as the fraction of incident radiation that is transmitted by the atmosphere. *τ*_sw_ is calculated using an elevation-based relationship:
    6 $$ {\tau}_{\mathrm{sw}}=0.75+2\times {10}^{-5}\times z $$
    where *z* is the elevation above sea level (m), which is of 32 m in the case of the official Montreal weather station (Pierre Elliott Trudeau (P-E-T)).
2. The NDVI (see Fig. 3) is the ratio of the differences in reflectivity between the near-infrared band (*ρ*_4_) and the red band (*ρ*_3_) to their sum:
  7 $$ \mathrm{NDVI} = \left({\rho}_4 - {\rho}_3\right)/\left({\rho}_4 + {\rho}_3\right) $$
  where *ρ*_3_ and *ρ*_4_ are the reflectivity for bands 3 and 4 of Landsat, respectively. The index produces values ranging from −1 to +1, where positive values indicate vegetated areas and where negative values represent non-vegetative surfaces, such as water, barren ground, and clouds.
3. Subsequently, the NDVI is used to generate the emissivity map (see Fig. 3). Various emissivity (*ε*) formulas are used depending on the NDVI values (Badarinath et al. 2005):

**Table:** 

• For NDVI values greater then 0.15 (NDVI > 0.15)	*ε* = 0.88
• For values 0.15 < NDVI < 0.7	*ε* = 1 + 0.05 ∗ ln(NDVI)
• For NDVI values > 0.7	*ε* = 0.985
• For values in *L*_*λband*4_ < 0.1	*ε* = 0.997 (water)
• For the area when NDVI < 0 and surface albedo (α) <0.047	*ε* = 0.99
4. Prior to estimating the surface temperature, the blackbody temperature (see Fig. 3) is calculated at satellite using the following equation:
  8 $$ Tb=\frac{k_2}{\left[ In\left(\frac{k_1}{L_{\lambda band6}}+1\right)\right]}-273.15 $$
  where *Tb* is the blackbody temperature at satellite in Celsius (Kelvin minus 273.15), *Lλ*_band6_ corresponds to Eq. 1 applied to band 6; the constant k_1_ (long-wave flux unit) is 607.76 (666.09) W/m^2^/sr/μm for TM (ETM+), and constant k_2_ is 1,260.56 K (1282.71) for TM (ETM+).
5. Lastly, the LST (see Fig. 3) is calculated using Eq. 9 below:
  9 $$ \mathrm{LST}=\frac{Tb}{1+\left(\uplambda \times \frac{Tb}{\gamma}\right)\times ln\varepsilon} $$
  where *λ* is the average of limiting wavelengths of band 6 of Landsat (*λ* = 11.5 μm), considering the following formula:
  $$ \gamma =\mathrm{h}\times \mathrm{c}/\mathrm{a}\left(=0.01438\kern0.5em \mathrm{m}\kern0.5em \mathrm{k}\right) $$
  where
  a: Boltzmann’s constant (1.38 × 10^−23^ J k)
  h: Planck’s constant (6.626 × 10^−34^ J s)
  c: Velocity of light (2.998 × 10^8^ m/s)

### Method to calculate the SIUHI threshold

A protocol was developed that seeks the most robust relationships or suitable identification approach for the occurrence and severity of SIUHIs between the different LST categories and the ambient and prevalent atmospheric variables (i.e., explicative co-variable) over the study region. Because solar radiation influences LST fluctuations (storage and release of heat during the day/night) through solar radiative incomes at the surface, this atmospheric variable was chosen as a predictor in the SIUHI identification protocol. To categorize the SIUHIs, the following five steps were used:

1. Creation of an urban mask within the city’s administrative boundaries that excludes agricultural fields and hydrography (see Study area);
2. Calculation of the mean LSTs for each image using the urban mask;
3. For each image, selection of pixel categories that exceeded the mean LST of the urban mask by +1, +2, +3, +4, +5, +6, and +7 °C. Note that an explanation is given in the following texts (i.e., in Montreal’s SIUHI categories and their identification) regarding the choice of these temperature thresholds;
4. Calculation of the hourly cumulative global solar radiation (GSR) observed at Montreal-Trudeau (i.e., P-E-T International Airport, see its location in Fig. 2) over the 48 h preceding image capture;
5. Determination of the linear relationship between the different SIUHI categories (i.e., related to various surface types) obtained from step 3 and the hourly cumulative GSR from step 4, using hourly Pearson-type correlation analysis.

## Results

### Montreal’s SIUHI categories and their identification

Satellite LST estimates are categorized using seven thermal thresholds in order to evaluate the link between various SIUHI categories and their evolution across the years, as well as the ambient and prevalent (24–48 h) solar radiation conditions. From the mean of each spatial LST for all images (after the application of the urban mask), we selected semi-subjectively (based on previous studies from Imhoff et al. (2010) and Singh and Bajwa (2007)) only those pixels warmer than the entire urban study area (Fig. 2) by 1 to 7 °C every degree (i.e., SIUHI + 1 to SIUHI + 7). Indeed, by relying on the spatial variability of all the 12 Landsat images, in terms of LST, there is virtually not a single hot pixel left represented beyond the highest threshold of 7 °C (see Table 3). Table 3 shows only the results (for brevity) for SIUHI + 3, SIUHI + 5, SIUHI + 6, and SIUHI + 7 corresponding to the total and percentage relative surface cover values (with respect to the total urban study area of 1420 km^2^) represented by each SIUHI category. Over time, the spread of SIUHIs has increased with the growth of the city; for example, SIUHI + 6 increased from 48.7 to 79.5 km^2^ from 1984 to 2008 for the study area, which correspond to the most compatible pair of images in terms of meteorological conditions, see Table 1. Although the meteorological conditions during Landsat’s morning overpasses were relatively windless (or with weak wind velocities) and sunny (see Table 1), the results show a broader range in the spatial extent of SIUHI, varying from 6 km^2^ for TM 1994 to 194.8 km^2^ in 1997 for the category SIUHI + 5 (see Table 3). The latter is characterized by LSTs higher than 26.9 °C (21.9 °C mean LST over the study area, i.e., for the SIUHI + 5 category in Table 3) for 13.7 % of the urban domain and by extreme SIUHI (SIUHI + 7) for 3.8 % of the same domain, despite the fact that the mean LST of TM 1997 is the coolest of all dates (see Table 3). Moreover, from 1984 to 2011, the mean LST of all images varies from 21.9 °C in TM 1997 to 30.7 °C in 2003, which does not correspond to the dates with the minimum/maximum values of total or relative surface cover by all SIUHI categories. In fact, the representation of SIUHI + 3 by TM 1997 is of 29.1 % compared to 26.5 % for TM 2003, which is characterized by hotter mean LST conditions. Thus, it is clearly necessary to consider meteorological conditions that occur before the satellite image is taken, in addition to the ambient global solar radiation (or other meteorological parameters) observed during the day of the satellite overpass.

**Table 3:** Four SIUHI categories for each Landsat image selected between 1984 and 2011 with their respective mean LST, standard deviation of mean LST, and total (km^2^) and relative (%) surface cover

	Mean LST of total study area (°C)	Standard deviation of mean LST	SIUHI + 3		SIUHI + 5		SIUHI + 6		SIUHI + 7	
Date (~10:15 a.m.)			Surface (km^2^)	% of study area	Surface (km^2^)	% of study area	Surface (km^2^)	% of study area	Surface (km^2^)	% of study area
July 10, 1984	27.5	3.7	372.3	26.2	105.4	7.4	48.8	3.4	16.2	1.1
June 17, 1987	23.1	3.8	407.3	28.7	104.0	7.3	39.6	2.8	13.0	0.9
July 17, 1989	28.1	3.8	353.0	24.8	109.4	7.7	40.4	2.8	21.7	1.5
July 25, 1992	26.6	3.8	376.7	26.5	94.9	6.7	50.7	3.6	19.4	1.4
June 29, 1994	24.1	2.4	177.7	12.5	6.0	0.4	1.5	0.1	0.5	0.0
June 28, 1997	21.9	4.4	413.5	29.1	194.8	13.7	101.9	7.2	54.6	3.8
June 11, 1999	28.6	3.8	341.7	24.0	113.1	8.0	43.1	3.0	19.9	1.4
June 8, 2001	27.5	4.6	438.7	30.9	191.5	13.5	113.9	8.0	66.3	4.7
July 15, 2003	30.7	4.1	377.2	26.5	122.4	8.6	69.0	4.9	29.1	2.0
June 27, 2005	29.6	3.6	355.1	25.0	88.8	6.2	37.7	2.7	20.1	1.4
July 5, 2008	29.4	4.3	393.2	27.7	134.3	9.4	79.6	5.6	33.2	2.3
July 14, 2011	29.3	3.9	456.2	32.1	130.9	9.2	53.6	3.8	29.7	2.1

Considering the total urban study area of 1420 km^2^

**Fig. 4:**
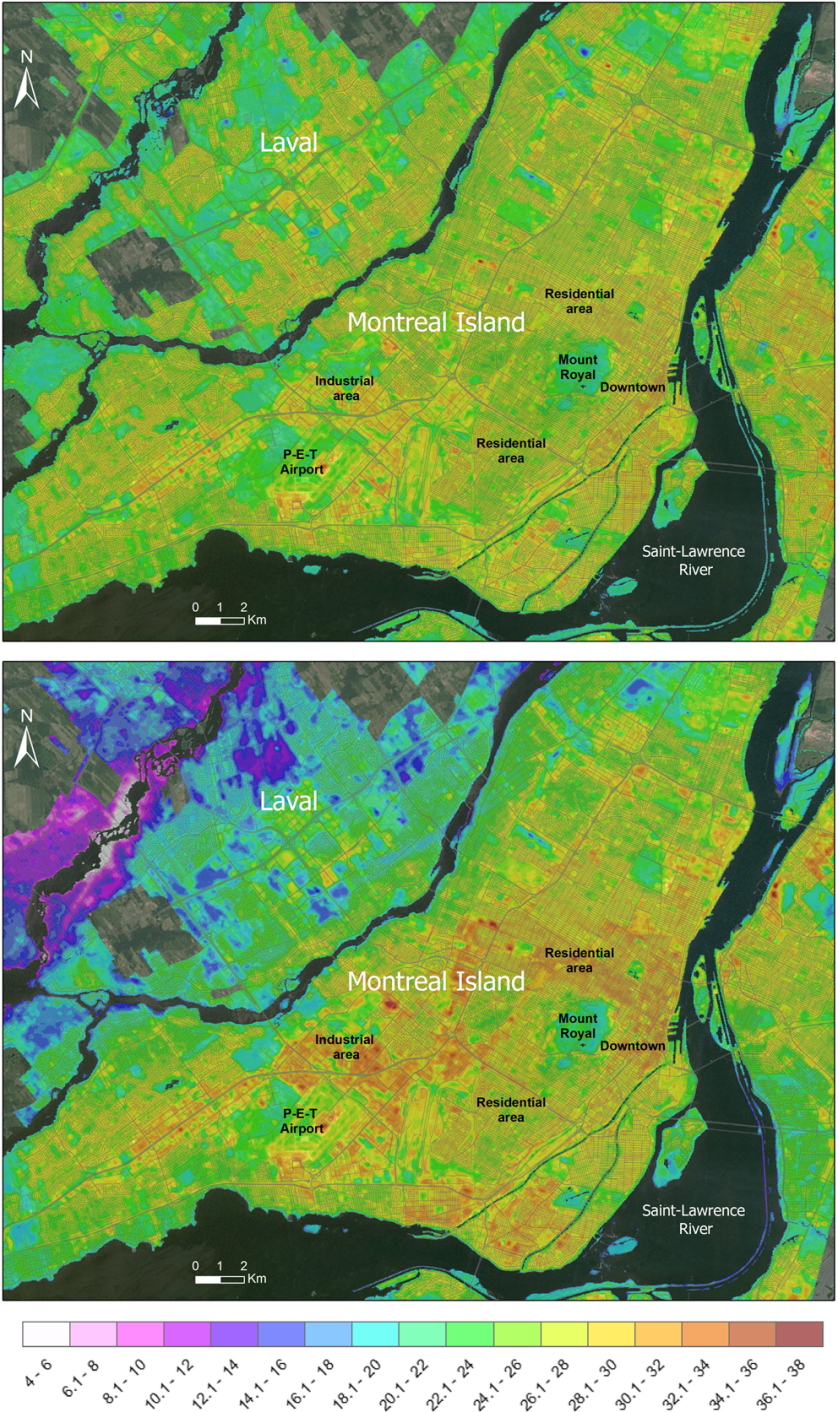
Land surface temperature (LST in °C) centered over the Montreal and Laval islands, for June 29, 1994 (*upper panel*) and June 28, 1997 (*lower panel*)

To illustrate the spatiotemporal heterogeneity in the formation of SIUHIs over the MMC, we used images of similar dates from 1994 and 1997 (June 29 and June 28, respectively) in Fig. 4 with a particular focus on the Montreal and Laval islands in order to examine the LSTs in detail. The two contrasting features between both dates indicate that the LSTs within the SIUHIs are more broadly distributed and higher across the area of interest in 1997 relative to 1994. In fact, as shown in Table 3, the standard deviation of mean LST was almost twice as high in 1997 as in 1994 (4.4 versus 2.4, respectively), suggesting a greater severity in SIUHI occurrences for certain residential and industrial areas in 1997 compared to 1994 (also visible in Fig. 4). However, in both cases, the daytime SIUHI patterns are strongly correlated with land use, with the warmest/coolest zones occurring over industrial/vegetated areas. When SIUHIs build up, residential areas can be very hot in the northeastern part of Mount Royal, but much cooler in the southwestern part (Fig. 4). As suggested by Roth et al. (1989), although SUHI (SIUHI in our case) intensities are greatest in the daytime and during the warm season, some meteorological factors must also be involved in their development (i.e., in terms of occurrence and severity).

### Appropriate predictors for SIUHI intensity

**Fig. 5:**
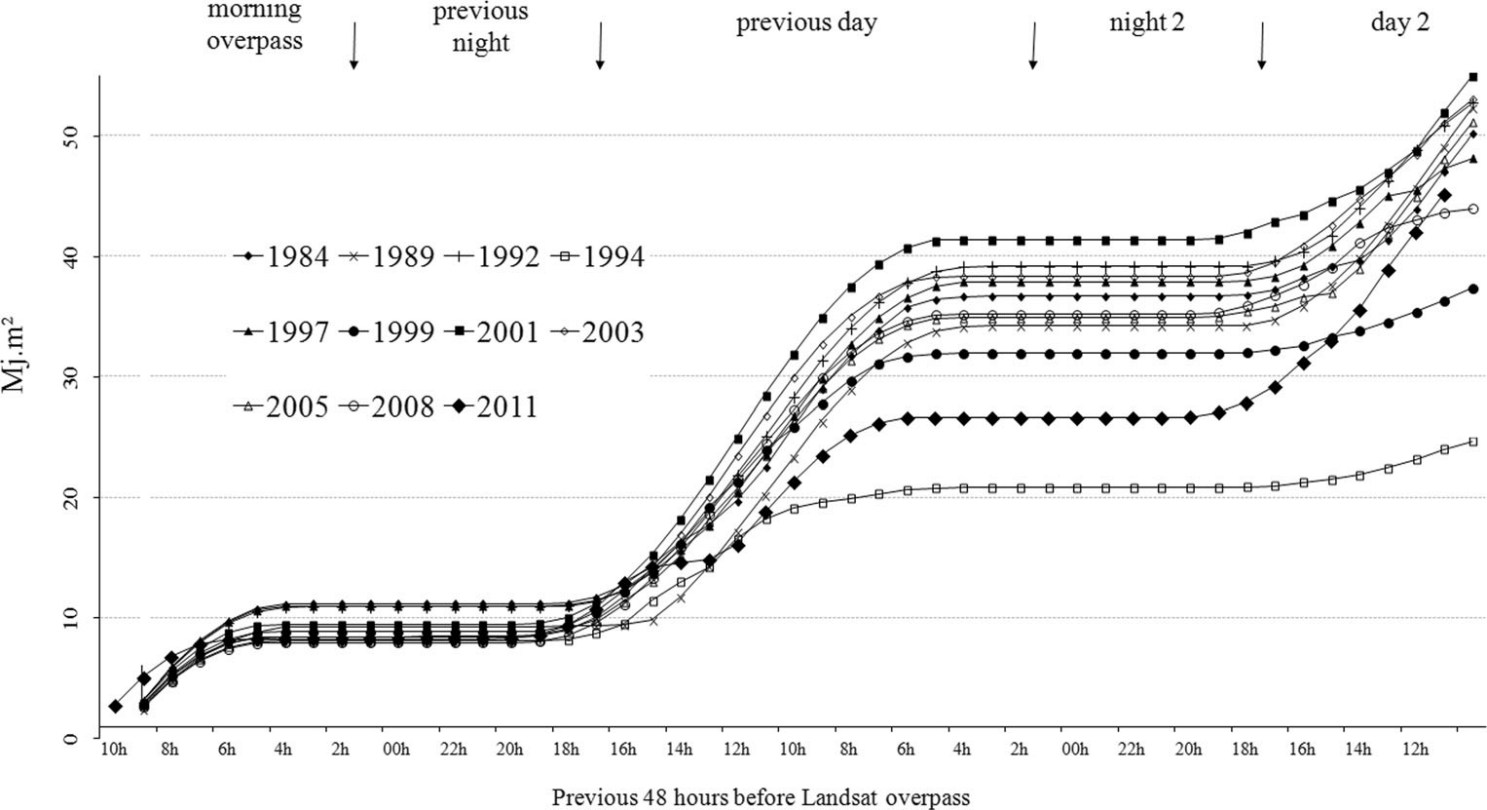
Hourly cumulative GSR for the 48 h before the overpass time for the acquisition of each of the 11 images

According to Hoffmann et al. (2011), CLUHI depends linearly on wind speed and relative humidity; however, the strongest relationship exists between urban–rural air temperature differences and cloud cover from the previous day. Clouds absorb and emit long-wave radiation, which reduces diurnal temperature variation (Oke 1987) and incoming shortwave radiation, thus reducing the amount of heat stored in urban materials (Hupfer and Kuttler 2006; Kawai and Kanda 2010). The most important factor for redistributing incident solar radiation, however, is urbanization related to land cover change. A higher level of latent heat exchange is associated with more vegetated areas, while the sensible heat exchange is more favored by built-up areas (Oke 1982). The different thermal capacities in absorbing and emitting heat among impervious surfaces, vegetation, soil, and water contribute significantly to the formation of SIUHI. In our case, the relation between the spatial extent and magnitude of the Montreal SIUHI and absorbed solar radiation by materials was obtained using observations taken from Environment Canada weather stations. To find the appropriate predictor, the relationship between the observed global solar radiation at Montreal P-E-T and the city’s SIUHI magnitude was analyzed (GSR is the direct and diffuse radiation received from a solid angle of 2 pi steradians on a horizontal surface, in MJ/m^2^). For all images, GSR observation data from the previous 48 h were extracted for all of Landsat’s overpass times using the Data Access Integration (DAI) portal, which is an online climatic and environmental data distribution tool (see http://loki.qc.ec.gc.ca/DAI/DAI-f.html). Weather station Montreal P-E-T (see its location in Fig. 2) was used for all images, with the exception of TM 1987, for which no GSR data had been recorded. Figure 5 shows the hourly cumulative GSR for all 11 images (i.e., except 1987) from the corresponding acquisition time (≈10 a.m.) and for the previous 24–48 h. These optimum GSR values represent the integrated irradiance for horizontal unobstructed surfaces over a 2-day period. The results indicate that some cumulative values can exceed 50 MJ/m^2^, while the minimum is close to 25 MJ/m^2^. Flat sequences of GSR correspond to nighttime periods or to times when no additional solar energy is received by instruments at the weather station. As shown in Fig. 5, the highest cumulative GSR over 48 h occurred during the years 2003, 2001, and 1992, and the lowest GSR values were recorded in 1999 and 1994. The latter year had the lowest effective radiative heating relative to all other years considered. In fact, the P-E-T weather station recorded an overcast sky on the previous day, confirming the impact of cloud cover (lower GSR) on the potential reduction of heat stored in urban materials during the 2 days prior to the satellite overpass.

**Fig. 6:**
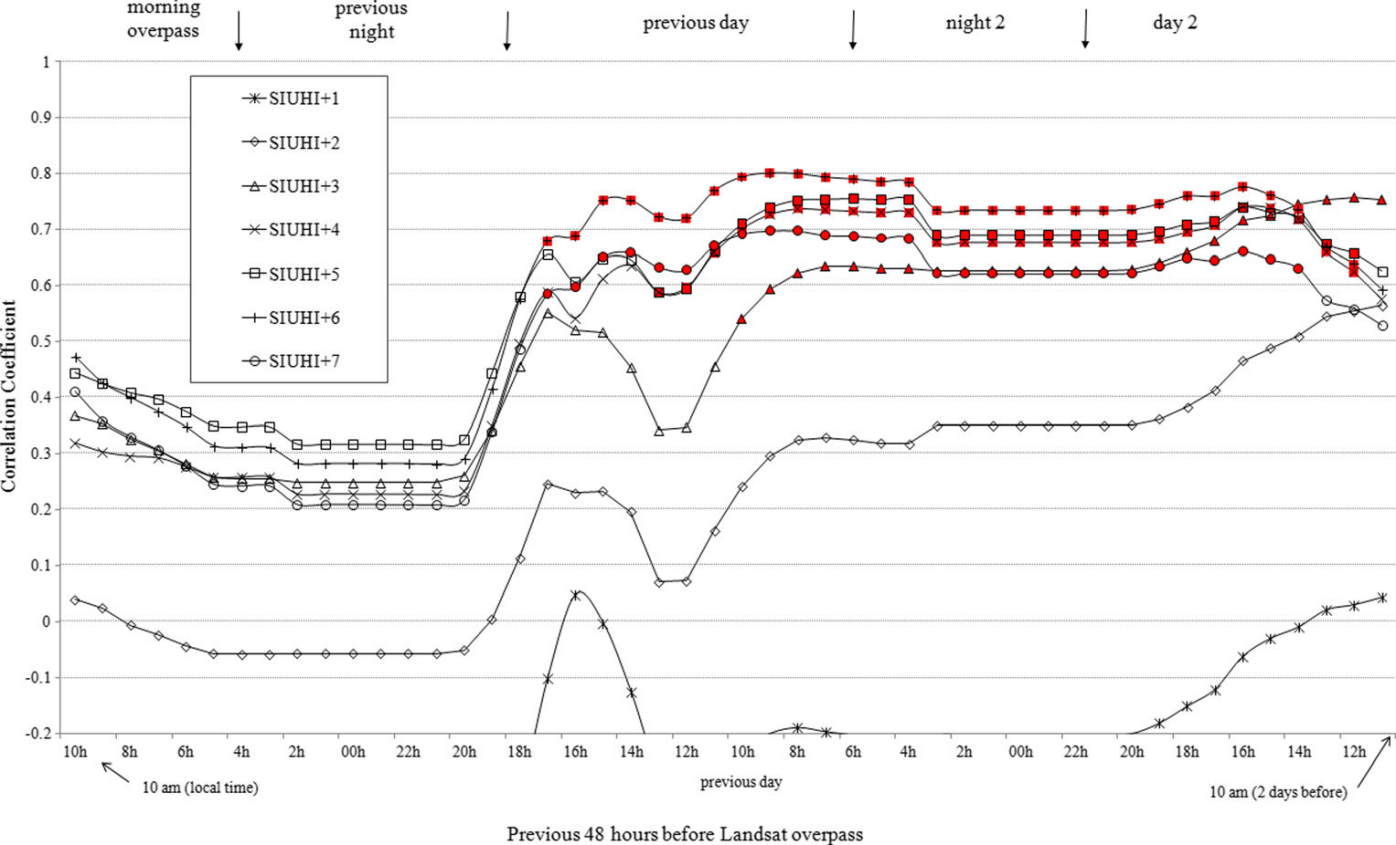
Pearson-type correlation between each SIUHI category for 11 images (as given in Table 3) and hourly cumulative GSR (as given in Fig. 5) during the previous 48 h of the satellite Landsat images. *Values in red* are statistically significant at the 95 % confidence level

Figure 6 represents the hourly correlation between each SIUHI category (Table 3) for all images and the corresponding cumulative GSR (Fig. 5) observed at the weather stations. The results suggest that cumulative GSR is highly significantly correlated (i.e., >0.6 at the 95 % confidence level) with SIUHI occurrence (i.e., SIUHI + 4 and above) from 17 to 48 h preceding the satellite image. Thus, what Landsat sees as a spatial pattern of upward thermal radiance received by its sensor corresponds to the cumulative effect of the atmospheric conditions (GSR in this case, received at the surface), i.e., the memory of radiative heat accumulated during the previous days. However, a higher correlation coefficient (0.8) between SIUHI occurrence and GSR is obtained 24 h before Landsat overpasses with the category SIUHI + 6. This high degree of linearity between the radiative predictor and SIUHI occurrence decreases slightly over time (i.e., up to 24 h before Landsat overpasses with the category SIUHI + 6) but still remains high (above 0.7) after 2 days. All correlations become significant from about 24 h before satellite overpasses for all SIUHI categories above +3 (i.e., statistically significant values at the 95 % confidence level; in red in Fig. 6). The other interesting characteristic of these relationships is that for SIUHI + 2, the correlations are low, while they are non-existent for SIUHI + 1. These results show that the memory of cumulative heating within the urban area is no longer effective for threshold categories of spatial SIUHI weaker than SIUHI + 3 and above. Thus, it takes approximately 2 days to warm the surface sufficiently for the material to accumulate this heating and to create favorable conditions for the development of a SIUHI, considering that all images are taken around 10 a.m., which does not necessarily correspond to the maximum amount of incoming energy. If the sunshine and clear sky conditions persist on the two consecutive days, more severe SIUHIs (i.e., SIUHI + 3 and above) start to develop with a more pronounced LST heterogeneity across the urban area. The 24 h prior to the occurrence of an SIUHI constitute the crucial period of time in which the sunny conditions will generate the difference between a day with homogenous LSTs and a day with more pronounced LST contrasts. In all cases and considering the highest correlations obtained, the SIUHI + 6 threshold within the study area can constitute the optimum criteria to characterize the newly defined surface urban heat island (i.e., without any rural reference), which can exacerbate local air temperature within the city and potentially have damaging effects on human health (Kovats and Hajat 2008). Furthermore, this indicator (SIUHI + 6) may help anticipate when possible heat alert thresholds could be reached locally under calm atmospheric conditions before they are observed at the weather station of reference (i.e., Montreal Airport). The links between LST and air temperature are discussed in the following section.

## Application for the heat health warning system

**Fig. 7:**
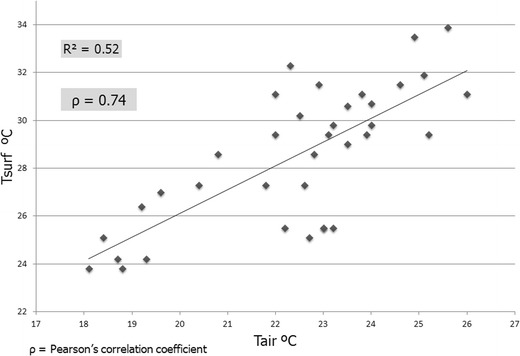
Remotely sensed Landsat LST of pixel locations versus air temperature observed at three official weather stations (i.e., *T*_air_ measured at 2-m height) during the satellite overpass time

Relationships between satellite-derived SUHIs and air temperature measured at 2 m remain empirical; to date, no simple general relation has been established (Voogt and Oke 2003). However, Klok et al. (2012) recently found a strong correlation (*R* ^2^ = 0.81) between Landsat LST and air temperature measured in situ, at satellite overpass time, in various locations at Rotterdam in the Netherlands (using four TM and ETM+ images during the warm season from 2005 to 2007). We conducted the same analysis as Klok et al. (2012) for our study area using 12 images of greater Montreal from 1984 to 2011. For the comparison between remotely sensed Landsat LSTs and in situ measured air temperature, three official meteorological stations operated by Environment Canada (i.e., P-E-T International Airport, Mirabel, and St Hubert, see their position in Fig. 2) are used. Measured air temperature at the moment of image acquisition were extracted and related to the corresponding surface temperature pixel value in a GIS. Results in Fig. 7 show a strong relationship between LST and air temperature, with a Pearson’s correlation coefficient (*ρ*) equal to 0.74 (statistically significant at the 99 % confidence level). These results concur with the work of Klok et al. (2012) for the Rotterdam area; the spatial variation in midmorning LST within the city of Montreal can be used as a strong indicator of variations in air temperature because a high LST clearly corresponds to a high overlying air temperature. This conclusion is reasonable because surface and air temperatures are linked through diabatic fluxes that, in turn, strongly influence the tendency of local air temperatures above the surface (i.e., through the thermodynamic relationship). In the absence of thermal advection (i.e., under calm or weak wind conditions, such as in our case), diabatic processes from both sensible heat and radiative fluxes, along with latent heat release from the ground, dominate the behavior and short-term fluctuation of local air temperature tendencies near the surface (without also non-significant vertical motion or turbulence within the boundary layer).

By analyzing the previous day’s atmospheric conditions, our results suggest that the cumulative amount of GSR favors the occurrence and severity of SIUHIs within the urban area of Montreal. Hence, weather services can use this information to anticipate the days on which SIUHIs will build up and during which heat alert thresholds could be reached in certain localized areas before a bulletin is launched for the entire metropolitan area (local excess in air temperature due to the relationship with the hot underlying surface). The actual Montreal heat plan is based on a threshold corresponding to a weighted average maximum air temperature (at 2-m height) of ≥33 °C over 3 days and a weighted average minimum temperature of ≥20 °C over three nights (Martel et al. 2010). These temperatures are currently being established at the airport station (Montreal P-E-T), which is used as a reference for the entire city, i.e., where the air temperature is not exacerbated by the surface underneath (or by local SIUHIs) or does not receive any urban influence at the microscale. This is not the case for LSTs observed or measured within the city, as mentioned above, where most of the population resides.

For the first time in Canada, on June 29, 2012, a severe weather bulletin issued by Environment Canada in Montreal regarding a high heat and humidity warning included the concept of UHIs (i.e., SIUHIs). It was specified that low-level air temperatures, as well as the residents’ discomfort levels, would be more acute in Montreal’s more densely urbanized areas, considering both the local exacerbating factors (i.e., land use) and prevalent/ambient solar radiation conditions. However, further exchanges between operational services and UHI specialists are needed to define a more accurate health warning system within the city. This would have the potential to improve the heat health watch and warning system (HHWWS) and its efficiency to substantially reduce the number of deaths or hospitalizations of vulnerable persons. Indeed, when combined with UHIs, heat waves under anticyclonic conditions with low or negligible wind speeds can result in large death tolls for the elderly and other highly vulnerable parts of the population within urban areas (Basara et al. 2010; Tan et al. 2010; Chebana et al. 2013).

## Summary and conclusions

Two factors led us to propose a new definition for SUHIs: the complexity of differentiating between what is urban and what is rural and the questionable relevance of using an external rural or non-urban reference to estimate UHI magnitude. The reference chosen for the newly defined SIUHI is the city’s mean LST for each of the 12 Landsat images taken during summer days between 1984 and 2011. Seven SIUHI categories were analyzed by considering GSR conditions prevailing prior to and during each acquisition date. The results revealed that cumulative GSR during the 2 days prior to acquisition of the image is a relevant predictor contributing to higher heat absorption in urban landscapes and influencing the occurrence and severity of SIUHIs (the highest correlation found on the previous day). The threshold of 6 °C above the city’s mean LST was found to be the optimum criterion among other thresholds to identify the newly proposed SIUHI. In 25 years, or between 1984 and 2008, the spread of SIUHI + 6 has increased by 63 % (or from 48.7 to 79.5 km^2^).

Our suggested method for defining SIUHIs could be replicated in other areas around the globe for which satellite and meteorological data are available. It is probable that the SIUHI threshold might be different for other cities with different satellite overpass times, sensors (Landsat 8, launched in February 2013), land use, or during another season. However, as a UHI is a relative measure that takes into account local physiographic and atmospheric conditions, the assessment of a hotspot (SIUHI) within a particular city needs to account for the cumulative effect of ambient and prevalent GSR as well as other meteorological conditions that could exacerbate local surface and air temperature conditions. As experienced in various cities across the world, the effects of SUHIs (SIUHIs in our case) have been successfully mitigated, as those have been clearly identified, by increasing urban vegetation and green spaces and by installing green roofs on the tops of buildings (Bass et al. 2002; Rosenzweig et al. 2006; Oberndorfer et al. 2007). Klok et al. (2012) found that a 10 % increase in green area decreased the surface temperature by 1.3 °C for the city of Rotterdam in the Netherlands.

The SIUHI + 6 can not only be considered as a local threshold for Montreal for identifying hotspots within the city but also be used as an important criterion to consider within an HHWWS. For example, Environment Canada’s meteorological services (Meteorological Service of Canada (MSC)) has used this criterion to better define and locate Montreal’s hottest areas during a heat spell and to determine where the help and intervention by public health services may need to be prioritized. The MSC is implementing a 2-year measurement campaign of air temperature within the urban core of Montreal during the 2013 and 2014 summer seasons. These campaigns are conducted using 30 sites identified on the basis of their specific fraction of vegetation and land cover. In the first phase, data from the sites will provide estimates of recurrent differences between the air temperature observed at the airport and air temperature observed in different areas within the city. Subsequent analysis will lead us to improve heat warning systems through spatial screening, allowing for timely warnings to be issued to the most vulnerable areas during a hot spell event. The threshold of 33 °C (over 3 days) can be reached in a residential area before it is reached at the airport or elsewhere in the city (SIUHI effects). In the second phase, a comparison will be made between LST and in situ measured air temperature to further analyze the relationship between both (surface and air) temperatures, based on the various urban materials and on the previous and ambient atmospheric conditions. This will help to further evaluate if the considered effects of ambient and prevalent GSR conditions on SIUHI thresholds induce similar behaviors on air temperature within the city, especially in terms of intensity and occurrence of the hottest conditions.

## Abbreviations

BLUHI: Boundary layer urban heat island
CLUHI: Canopy-layer urban heat island
DAI: Data Access Integration
DN: Digital number
ETM+: Enhanced Thematic Mapper Plus
GIS: Geographical information system
GSR: Global solar radiation
HHWWS: Heat health watch and warning system
k_1_: Calibration constants (TM = 607.76 or ETM+ = 666.9)
k_2_: Calibration constants (TM = 1260.56 or ETM+ = 1282.71)
L: Spectral radiance
LST: Land surface temperature
MJ: Megajoule (equal to one million (10^6^) joules)
MMC: Montreal Metropolitan Community
MSC: Meteorological Service of Canada
NDVI: Normalized Difference Vegetation Index
NIR: Near infrared
P-E-T: Pierre Elliott Trudeau
RBL: Rural boundary layer
SD: Standard deviation
SEBAL: Surface Energy Balance Algorithms for Land
SIUHI: Surface intra-urban heat island
SSUHI: Subsurface urban heat island
SUHI: Surface urban heat island
TCZ: Thermal climate zone
TM: Thematic Mapper
TOA: Top of atmosphere (albedo)
UBL: Urban boundary layer
UCL: Urban canopy layer
UCZ: Urban climate zone
UHI: Urban heat island
USGS: US Geological Survey

## Footnotes

1 SEBAL; Surface Energy Balance Algorithms for Land. Idaho Implementation. Advanced Training and Users Manual. August, 2002. Version 1.0 (http://www.dca.ufcg.edu.br/mwg-internal/de5fs23hu73ds/progress?id=zBkOEDSbPr)
